# Prognostic impact of coronary microvascular dysfunction assessed by caIMR in overweight with chronic coronary syndrome patients

**DOI:** 10.3389/fendo.2022.922264

**Published:** 2022-08-10

**Authors:** Cailin Feng, Fuad A. Abdu, Abdul-Quddus Mohammed, Wen Zhang, Lu Liu, Guoqing Yin, Yundi Feng, Ayman A. Mohammed, Redhwan M. Mareai, Xian Lv, Tingting Shi, Yawei Xu, Xuejing Yu, Wenliang Che

**Affiliations:** ^1^ Department of Cardiology, Shanghai Tenth People’s Hospital, Tongji University School of Medicine, Shanghai, China; ^2^ RainMed Medical, Suzhou, China; ^3^ Department of Cardiology, Shanghai Tenth People’s Hospital Chongming Branch, Shanghai, China

**Keywords:** coronary microvascular dysfunction, coronary angiography-derived index of microcirculatory resistance, overweight, chronic coronary syndrome, clinical outcomes

## Abstract

**Objective:**

Coronary microvascular dysfunction (CMD) may associate with adverse cardiovascular events in obese patients. Coronary angiography-derived index of microcirculatory resistance (caIMR) is proposed as a less-invasive and pressure-wire-free index to assess CMD. We aimed to investigate the impact of coronary microvascular function assessed by caIMR in patients with overweight and chronic coronary syndrome (CCS).

**Methods:**

CCS patients who underwent coronary angiography between 2015 to 2018 were included. Overweight was defined as BMI≥24.0kg/m². Impaired coronary microvascular function was defined as caIMR≥25U. The patients were classified according to BMI and caIMR. The primary endpoint was major adverse cardiac events (MACE). Kaplan-Meier and Cox regression analyses evaluated the association between caIMR and MACE.

**Results:**

Two hundred and eighty-two CCS patients were enrolled. Among these, 169 (59.93%) were overweight. Impaired coronary microvascular function was higher in overweight patients than in patients with normal weight (49.70% vs. 38.05%; P=0.035). During 35 months of follow-up, 33 MACE had occurred. Among the total CCS population, MACE was higher in patients with high caIMR than in low caIMR (18.11% vs. 6.45%, P=0.003). In subgroups analysis, MACE was higher in overweight patients with high caIMR than low caIMR (20.24% vs. 7.06%, P=0.014), while there were no significant differences in normal-weight patients. Multivariate Cox analysis demonstrated that caIMR≥25 was independently associated with MACE in overweight patients (HR, 2.87; 95% CI, 1.12-7.30; P=0.027) but not in the normal-weight patients. In addition, caIMR showed a significant predictive value for adverse outcomes in overweight patients and provided an incremental prediction when added to a prediction model with BMI.

**Conclusions:**

Impaired coronary microvascular function assessed by caIMR was common and is an independent predictor of MACE in overweight patients with CCS.

## Introduction

Obesity is one of the ongoing epidemics worldwide; the Global Burden of Disease group estimated that about 4 million deaths worldwide were related to high body mass index (BMI) (1). Obesity has been linked to chronic metabolic disorders such as coronary heart disease (CAD), hypertension, hyperlipidemia, diabetes mellitus, and various malignancies, resulting in poor clinical outcomes (2–6). The pathogenesis principles responsible for increased risk in obesity are complicated, including increased oxidative stress, enhanced sympathetic nervous system activity, and low-grade systemic inflammation, as they can lead to coronary microvascular dysfunction (CMD), which is, in turn, associated with increased adverse clinical outcomes (7–9).

CMD is recognized as increased blood flow in response to stress due to increased microvascular resistance or impaired microvascular dilatation, resulting in myocardial ischemia and increased cardiovascular risk (10). Clinical studies have found that CMD is more common and may associate with adverse cardiovascular events in obese individuals (8, 11, 12). A recent study showed that obese patients with CMD assessed by coronary flow reserve (CFR) have a 2.5-fold higher risk of developing clinical adverse events (11). This may imply a potential correlation between obesity, CMD, and poor clinical outcomes.

The evaluation of coronary microvascular function using invasive wire-derived techniques is predominantly governed by CFR and the index of microcirculatory resistance (IMR). The clinical utility of CFR to evaluate coronary microvascular function among obese patients has been shown in prior investigations, which reported that obese patients are associated with impaired CFR (8, 11). In contrast to CFR, IMR is more specific to the microvasculature, more quantitative, and is not influenced by hemodynamic changes or epicardial stenosis (13). IMR has previously been proven to have significant diagnostic and prognostic performance in various clinical disorders (14–16). However, owing to its invasive nature, longer procedure duration, higher cost, technical complexity, and the usage of adenosine to achieve maximal hyperemia, its use in clinical practice remains incredibly limited. Recently developed functional coronary angiography parameters enable simpler, rapid, and do not require pressure wire or adenosine, using coronary angiography derived index of microvascular resistance (caIMR) (17). Several clinical studies investigated the role of caIMR and concluded that caIMR is a valuable predictor and has a high diagnostic performance in different clinical settings, including coronary artery disease patients who received the percutaneous coronary intervention (PCI), ST-Segment elevation myocardial infarction (STEMI), and non-obstructive coronary arteries (18–21). Notably, there have been no investigations into the prognostic value of the IMR either invasively or non-invasively in obese patients.

Therefore, this study aimed to clarify the prognostic impact of coronary microvascular function derived by caIMR in overweight with chronic coronary syndrome (CCS) patients.

## Methods

### Study population

Between June 2015 and May 2018, CCS patients who have undergone coronary angiography for suspected angina at Shanghai Tenth People’s Hospital were enrolled in this single-center retrospective observational study.

The inclusion criteria of the present study were (1): Age >18 years old (2); Patients comply with the 2019 ESC guidelines for the diagnosis and management of CCS (22). Patients were excluded if they had a myocardial infarction (MI) within the previous 7 days, severe coagulopathy or bleeding disorders, malignant tumor and estimated life span less than one year, left ventricular ejection fraction<35%, and post coronary artery bypass graft surgery. The caIMR exclusion criteria include: poor contrast opacification, serious vascular overlap or distortion of the target vessel, and poor angiographic image quality are insufficient to demonstrate the contour detection requested by the FLASH software.

The basic clinical information, past medical history, and laboratory data of all subjects in this study were documented. Electrocardiograms, echocardiograms and coronary angiography information were collected from all participants.

The study protocol was approved by the ethics committee of Shanghai Tenth People’s Hospital and was performed in compliance with the Declaration of Helsinki. Written informed consent was obtained from all patients.

### caIMR measurement

caIMR was achieved using software with the Flash Angio system (including the Flash Angio console, Flash Angio software, and Flash Pressure transducer; Rainmed Ltd., Suzhou, China). The theory and details of the measurement of caIMR were reported by Yong Huo’s team (17), as computed as follows: $\text{caIMR}=\;Pd_{hyp}\frac{L}{K\cdot V_{diastole}}$ caIMR is the measurement of pressure at maximal hyperemia as described by the previous study (17); the microvascular resistance and flow velocity are proportional to that of hyperemia in diastole and are computed in a unit volume of myocardium distal to the L position at maximum hyperemia. caIMR was achieved by following three steps. A; Construction of a 3D mesh network along the coronary vessel. B; The hyperemic aortic pressure is based on mean arterial pressure (MAP) at angiography; MAP x 0.2 when MAP≥95 mmHg or MAP – MAP x 0.15 when MAP< 95 mmHg. C; The computation of caIMR using the above equation. In the equation above, (Pd)hyp means the mean pressure (unit: mmHg) at the distal position at the maximal hyperemia, and L is a constant (non-dimensional) that mimics the length from the inlet to the distal position (L=75, mimicking 75 mm downstream from the inlet of coronary arterial tree), K is a constant (K=2.1) obtained from previous literature, Vdiastole is the mean flow velocity (unit: mm/s) at the distal position at diastole and Vhyp = K · V_diastole_, refers to the mean flow velocity (unit: mm/s) at the distal position at the maximal hyperemia.

A total of 282 patients were measured for caIMR. The caIMR was measured in 312 stenotic epicardial arteries. Those who underwent PCI had their caIMR measured after the PCI procedure. If a patient had multiple coronary stenosis lesions, the highest caIMR value was used. caIMR measurement was accomplished by 2 experienced cardiologists without any awareness of experiment outcomes.

### Definitions and outcomes

In the present study, BMI ≥24.0kg/m² was applied to define overweight in Chinese populations, as recommended by the guidelines for prevention and control of overweight and obesity in Chinese adults (23). Diabetes was defined as following (1): HbA1c ≥ 6.5 % (≥ 48 mmol/mol) (2); Random plasma glucose ≥ 200 mg/dl (≥ 11.1 mmol/l) (3); Fasting plasma glucose ≥ 126 mg/dl (≥ 7.0 mmol/dl); and (4) OGTT 2−hour glucose in venous plasma ≥ 200 mg/dl (≥ 11.1 mmol/l) (24). Hyperlipidemia is defined as: fasting serum LDL-cholesterol of ≥3.37 mmol/L (130 mg/dL), HDL-cholesterol of ≤1.04 mmol/L (40 mg/dL), triglyceride of ≥1.76 mmol/L (150 mg/dL), and/or total cholesterol ≥5.18mmol/L (200mg/dL). Hypertension was defined as systolic blood pressure ≥140 mmHg or diastolic blood pressure ≥90 mmHg. Impaired coronary microvascular function was defined as caIMR ≥25U, according to the established cut-off value (17).

To investigate the impact of caIMR, patients were classified into an overweight group (BMI ≥24.0kg/m²) and a normal-weight group (BMI<24.0kg/m²), and each of these groups was further classified into low and high caIMR groups according to the cut-off value of 25U (17).

The follow-up information was recorded by two trained cardiologists at Shanghai Tenth People’s Hospital by collecting medical and surgical records. All patients were followed up for 35 months through phone calls and outpatient visits. The primary clinical endpoints were the first major adverse cardiac events (MACE), including cardiovascular death, nonfatal MI, heart failure, and ischemia-driven revascularization. We defined cardiovascular death as death due to malignant arrhythmia, myocardial infarction, or refractory heart failure. Nonfatal MI was defined as the presence of typical symptoms of myocardial ischemia and observed dynamic variation of myocardial enzymes except death. Heart failure was defined as hospitalization due to acute or chronic heart failure in accordance with 2021 ESC guidelines for the diagnosis and treatment of acute and chronic heart failure (25). Ischemia-driven revascularization means the revascularization procedure which was caused by a positive test or the replace of angina pectoris.

### Statistical analysis

The Statistical Package for Social Sciences (SPSS), version 22, was used to analyze the data of this study. GraphPad software, version 8.0.1, was used to create the figures. The data were recorded as a percentage (%) for categorical variables and mean ± standard deviation (SD) or medians and interquartile ranges for continuous variables. The independent sample t-test or Mann–Whitney U test was used to compare numerical variables between groups. For categorical variables, the chi-square test and Fisher’s exact tests were used as appropriate. Kaplan-Meier analysis was used to compute MACE-free survival rates, and differences between the groups were assessed by the log-rank test. Cox regression models were used to derive adjusted hazard ratio (HR) to evaluate predictors of the clinical endpoints. The univariate analysis includes cardiac risk variables and parameters that were statistically significant at baseline between groups. Factors that were univariate predictors with P< 0.10 were incorporated into the multivariable models. Values are expressed as hazard ratio (HR) with a 95% confidence interval (CI). To assess the effect of caIMR on MACE between overweight and normal-weight groups, an interaction analysis was performed using a Cox regression model. The receiver operating characteristic (ROC) curve clarifies the prognostic value of caIMR in the overweight group. A further assessment of the incremental prognostic value of caIMR using integrated discrimination improvements (IDI) and net reclassification improvements (NRI) was performed. All the tests were two-sided, and a P-value< 0.05 was considered statistically significant.

## Result

### Baseline characteristics

**Figure 1:**
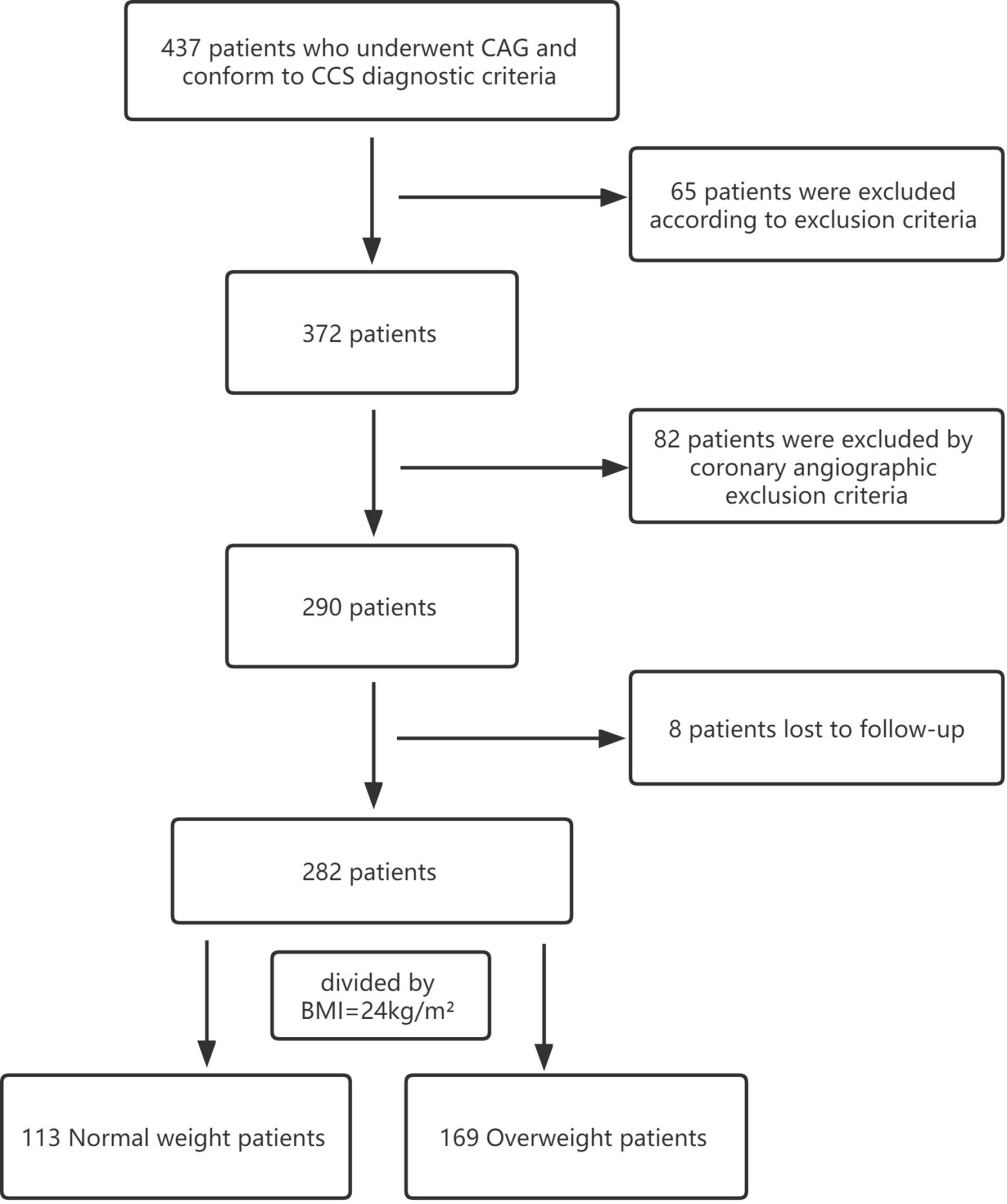
Flowchart of patients enrolled. CAG, coronary angiography;CCS, chronic coronary syndrome; BMI, body mass index.

Baseline, biochemical and angiographic characteristics of the study population are shown in **Tables 1**, **2**. Two hundred and eighty-two CCS patients were enrolled in the final analysis of the present study, with a mean age of 64.95 ± 9.46 years. Among these, 169 (59.93%) patients had overweight, while 113 (40.07%) were of normal weight (**Figure 1**). Compared to the normal weight group, patients in the overweight group had a higher rate of male patients, diabetes mellitus, systolic blood pressure, diastolic blood pressure, triglycerides, and HbA1c, while patients in the normal weight were older. The proportion of impaired coronary microvascular function (caIMR≥25) was higher in overweight patients than in patients with normal weight (49.70% vs. 38.05%; P=0.035). The distribution of other demographic, baseline information, and angiographic characteristics were similar between the two groups.

**Table 1:** Baseline characteristics.

	Normal weight (n = 113)	Overweight (n = 169)	P-value
**Demographics**			
Age (years)	66.42 ± 9.22	63.97 ± 9.51	0.033
Male, n (%)	66 (58.41)	129 (76.33)	0.002
**Cardiovascular risk factors**			
Diabetes history	29 (25.66)	67 (39.64)	0.015
BMI	22.49 (21.33-23.28)	26.45 (25.08-27.98)	<0.01
Hypertension history	74 (65.49)	125 (73.96)	0.126
Hyperlipidemia history	19 (16.81)	40 (23.67)	0.166
Atrial fibrillation history	5 (4.42)	9 (5.33)	0.787
Post-PCI	90 (79.65)	131 (77.51)	0.670
Smoking history	23 (20.35)	45 (26.63)	0.228
Family history of CAD	4 (3.54)	8 (4.73)	0.768
LVEF	63.00 (60.00-65.00)	63.00 (60.00-65.00)	0.899
**Vital signs**			
Systolic blood pressure (mmHg)	130.11 ± 21.80	136.71 ± 20.30	0.010
Diastolic blood pressure (mmHg)	73.31 ± 11.75	78.09 ± 11.63	0.001
Heart rate	73.00 (68.00-80.00)	76.00 (68.50-82.00)	0.144
**Biochemical parameters**			
TG (mmol/L)	1.19 (0.95-1.69)	1.46 (1.15-2.31)	<0.01
TC (mmol/L)	3.47 (2.98-4.14)	3.56 (2.93-4.28)	0.759
LDL-C (mmol/L)	1.71 (1.40-2.28)	1.79 (1.34-2.41)	0.746
HbA1c (g/L)	6.00 (5.65-6.75)	6.30 (5.80-7.10)	0.002

Values are shown as n (%), mean ± SD, or medians (interquartile ranges). BMI: body mass index. PCI: percutaneous coronary intervention; LVEF: left ventricular ejection fraction; TG: triglycerides. TC: total cholesterol; LDL-C: high-density lipoprotein; HbA1c: glycated hemoglobin.

**Table 2:** Coronary and angiographic characteristics.

	Normal weight(n = 113)	Overweight(n = 169)	P-value
**Coronary Physiological parameters**			
caFFR	0.92 (0.91-0.95)	0.93 (0.90-0.95)	0.792
caIMR	23.80 (19.10-30.75)	24.70 (19.25-34.50)	0.380
caIMR≥25U	43 (38.05)	84 (49.70)	0.035
**Target vessel**	^*^n=123	^*^n=189	–
LAD, n (%)	74 (60.16)	126 (66.67)	0.242
LCX, n (%)	23 (18.70)	22 (11.64)	0.083
RCA, n (%)	26 (21.14)	41 (21.69)	0.907

Values were shown as n (%) or medians (interquartile ranges). caFFR: coronary angiography−derived fractional flow reserve. caIMR: coronary angiography−derived index of microcirculatory resistance; LAD: left anterior descending; LCX: left circumflex; RCA: right coronary artery. ^*^ n of target vessel is total number of left anterior descending = 200, left circumflex = 45 and right coronary artery = 67.

Three hundred and twelve coronary arteries were assessed in this study: left anterior descending artery (LAD), n = 200 (64.10%); left circumflex artery (LCX), n = 45 (14.42%); and right coronary artery (RCA), n = 67 (21.47%).

### Clinical outcomes

**Figure 2:**
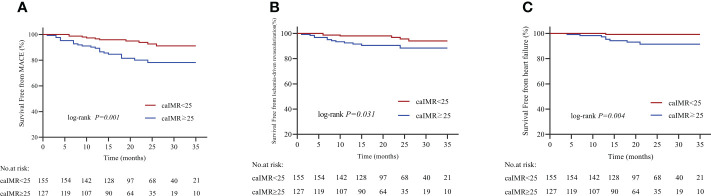
**(A)** Kaplan-Meier survival curves for MACE in CCS patients with high caIMR vs. low caIMR. **(B)** Kaplan-Meier survival curves for ischemia-driven revascularization in CCS patients with high caIMR vs. low caIMR. **(C)** Kaplan-Meier survival curves for heart failure in CCS patients with high caIMR vs. low caIMR. MACE, major adverse cardiovascular events; caIMR, coronary angiography−derived index of microcirculatory resistance.

**Figure 3:**
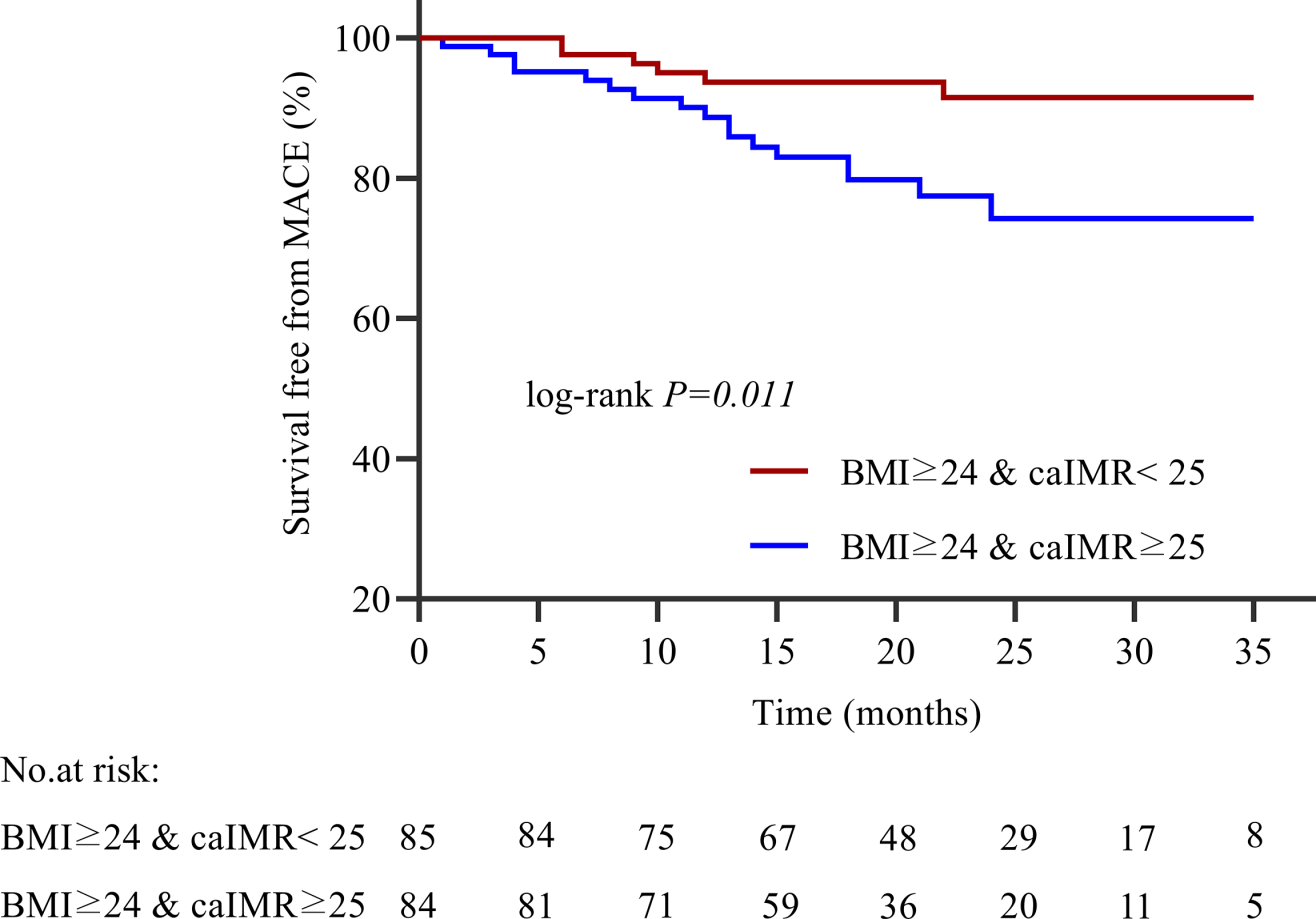
Kaplan-Meier survival curves for MACE in overweight patients with high caIMR vs. low caIMR. caIMR: coronary angiography−derived index of microcirculatory resistance; BMI: body mass index.

**Figure 4:**
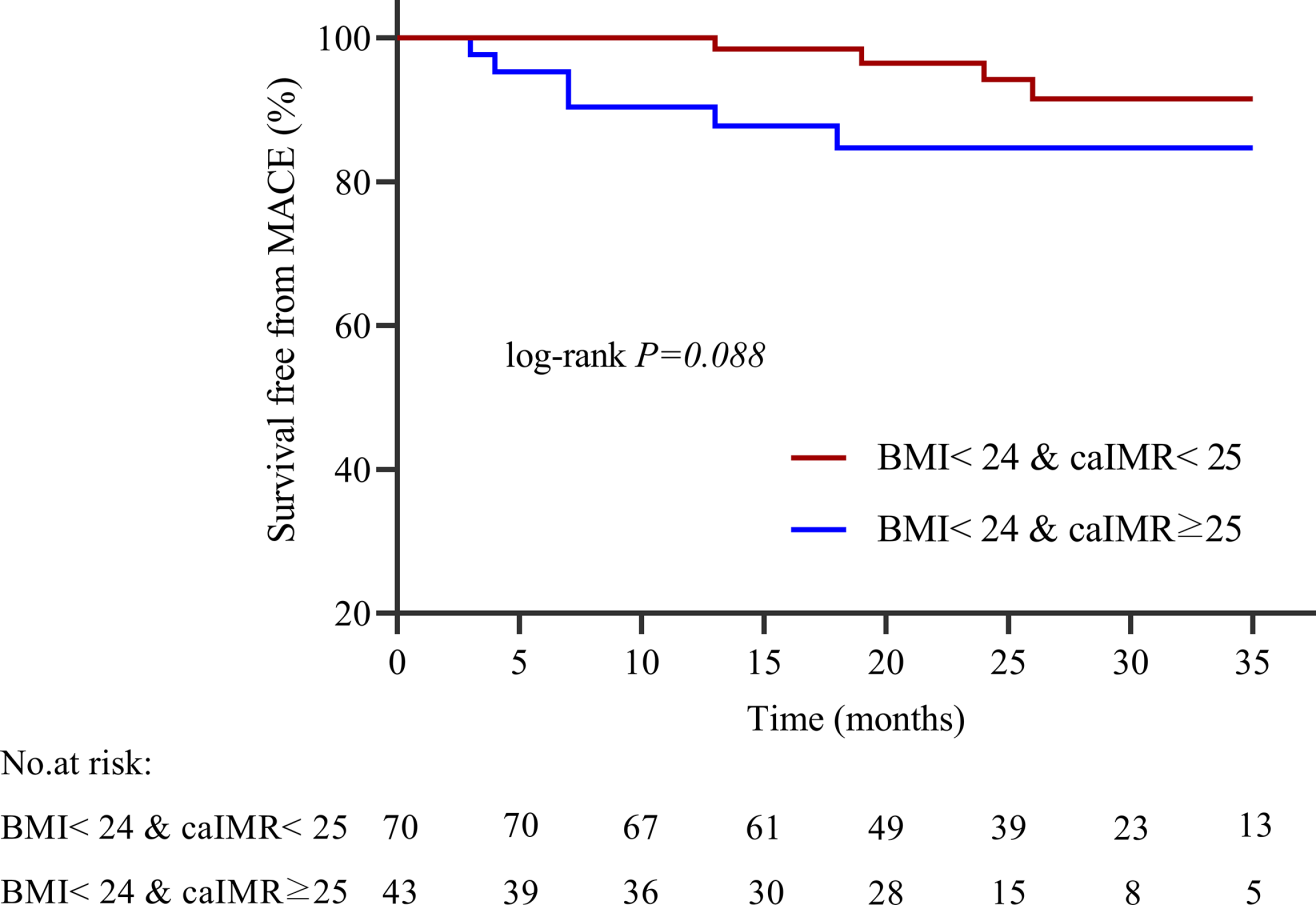
Kaplan-Meier survival curves for MACE in normal weight patients with high caIMR vs. low caIMR. caIMR, coronary angiography−derived index of microcirculatory resistance; BMI, body mass index.

During 35 months of follow-up, a total of 33 MACE have occurred. The incidence rate of MACE was higher in the high caIMR group than in the low caIMR group (18.11% vs. 6.45%, P=0.003). In subgroups analysis, the more apparent differences only appear in the overweight group with high caIMR; that is, the rate of MACE in the overweight patients with high caIMR is higher than in the low caIMR patients (20.24% vs. 7.06%, P=0.014). However, there were no differences in the normal weight group between high and low caIMR patients (13.95% vs. 5.71%, P=0.176) (**Table 3**). Through survival-free analysis of composite MACE, the Kaplan-Meier curves display that patients with high caIMR had a significantly increased risk of MACE than low caIMR in total populations (log-rank P=0.001) (**Figure 2A**). Likewise, when only ischemia-driven revascularization and heart failure were analyzed separately, patients with high caIMR still had a higher risk of MACE than low caIMR patients (log-rank P = 0.031, and log-rank P=0.004, **Figures 2B, C**). The same results were only noted among high and low caIMR in overweight patients (log-rank P=0.011) (**Figure 3**). However, this difference was not apparent among the normal-weight group (log-rank P=0.088) (**Figure 4**).

**Table 3:** Clinical Outcomes by caIMR and BMI.

	Total			Normal weight			Overweight		
	caIMR< 25 (n = 155)	caIMR ≥ 25 (n = 127)	P-value	caIMR< 25 (n = 70)	caIMR ≥ 25 (n = 43)	P-value	caIMR< 25 (n = 85)	caIMR ≥ 25 (n = 84)	P-value
MACE	10 (6.45)	23 (18.11)	0.003	4 (5.71)	6 (13.95)	0.176	6 (7.06)	17 (20.24)	0.014
Cardiac death	1 (0.65)	0	1.000	0	0	–	1(1.18)	0	1.000
Revascularization	6 (3.87)	12 (9.45)	0.084	2 (2.86)	3 (6.98)	0.367	4(4.71)	9 (10.71)	0.161
Nonfatal MI	2 (1.29)	3 (2.36)	0.660	1 (1.43)	0	1.000	1(1.18)	3 (3.57)	0.368
Heart failure	1 (0.65)	8 (6.30)	0.012	1 (1.43)	3 (6.98)	0.153	0	5 (5.95)	0.029

Values were shown as n (%). MACE, major adverse cardiac events; caIMR, coronary angiography-derived index of microcirculatory resistance; MI, myocardial infarction.

### Outcome predictors

**Tables 4**, **5** displayed the impact of potential risk factors on the risk of MACE across subgroups through univariate and multivariate analyses. Univariate Cox regression analysis identified caIMR≥25 as a significant predictor of MACE in total CCS patients (HR, 3.19; 95% CI, 1.52-6.71; P=0.002). It is worth noting that this relationship was only found in patients with overweight rather than normal-weight patients (HR, 3.16; 95% CI, 1.24-8.01; P=0.016). Multivariate Cox regression analysis showed that caIMR≥25 is still independently associated with an increased hazard of MACE in patients with overweight even after adjusting for other confounding risk factors (HR, 2.87; 95% CI, 1.12-7.30; P=0.027). Notably, there was a significant effect of the interaction term of calMR ≥25 in predicting MACE between overweight and normal-weight groups (P = 0.002 for interaction). Diabetes was also a predictor of MACE, either in univariate or multivariate analysis in our study.

**Table 4:** Univariate analysis for MACE.

	Total		Normal weight		Overweight	
	HR (95% CI)	P	HR (95% CI)	P	HR (95% CI)	P
Age	1.02 (0.98-1.06)	0.313	1.02 (0.95-1.10)	0.606	1.00 (0.96-1.05)	0.348
Male	2.18 (0.90-5.29)	0.084	3.11 (0.66-14.66)	0.152	1.54 (0.52-4.53)	0.725
Diabetes	2.68 (1.35-5.35)	0.005	4.48 (1.26-15.87)	0.020	2.79 (1.18-6.61)	0.019
SBP	1.00 (0.99-1.02)	0.803	0.99 (0.97-1.02)	0.617	1.00 (0.98-1.02)	0.714
DBP	1.00 (0.97-1.03)	0.831	0.97 (0.92-1.02)	0.270	1.00 (0.97-1.04)	0.900
TG	0.88 (0.61-1.27)	0.494	0.61 (0.17-2.24)	0.456	0.86 (0.58-1.29)	0.466
HbA1c	0.99 (0.75-1.30)	0.919	0.62 (0.39-1.74)	0.615	0.98 (0.72-1.32)	0.877
caIMR≥25	3.19 (1.52-6.71)	0.002	2.87 (0.81-10.19)	0.103	3.16 (1.24-8.01)	0.016

Univariate analysis enrolled factors with a P-value< 0.05 in the baseline. caIMR, coronary angiography−derived index of microcirculatory resistance; SBP, systolic blood pressure; DBP,diastolic blood pressure; TG, triglycerides; HbA1c, glycated hemoglobin; HR, hazard ratio; CI, confidence interval.

**Table 5:** Multivariate analysis for MACE.

	Total		Normal weight		Overweight		
	HR (95% CI)	P-value	HR (95% CI)	P-value	HR (95% CI)		P-value
caIMR≥25	2.75 (1.29-5.85)	0.009	–	0.285	2.87 (1.12-7.30)		0.027
Diabetes	2.23 (1.10-4.49)	0.025	4.48 (1.26-15.87)	0.020	2.51 (1.06-5.96)		0.037

Multivariate analysis adjusted for factors with a P-value< 0.1 in univariate analysis. caIMR, coronary angiography−derived index of microcirculatory resistance; HR, hazard ratio; CI,confidence interval.

### The incremental predictive value of caIMR

**Figure 5:**
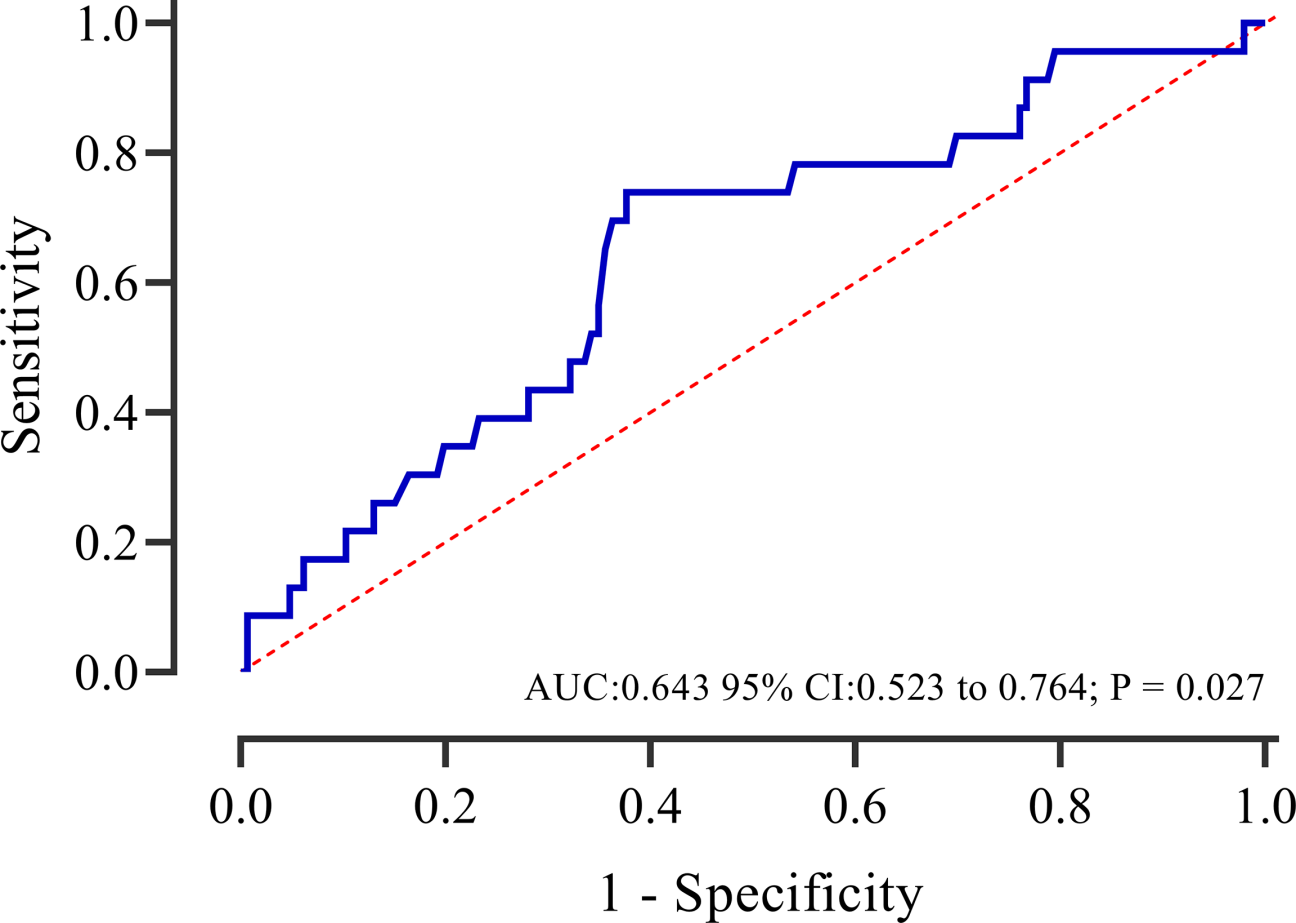
Receiver operating characteristic curves of caIMR for predicting adverse events in overweight patients. AUC, area under thecurve; CI, confidence interval.

The ROC curve analysis showed that caIMR could provide a significant predictive value for MACE in patients with overweight, with a sensitivity of 0.739 and specificity of 0.623 (95% CI 0.523-0.764, P = 0.027) (**Figure 5**). Additional analysis was performed to examine whether caIMR had an incremental predictive ability for MACE in overweight patients (**Table 6**). The results showed that caIMR, when added to a model with BMI, the combined model was able to predict adverse outcomes with more accuracy and provided significant improvement in the IDI (4.3, 95% CI: 0.1-12.3, p = 0.020) and NRI (32.8, 95% CI: 0.0-55.4, p = 0.020).

**Table 6:** Model improvement for the caIMR in combination with BMI.

	AUC (95% CI)	IDI (95% CI)	p value	NRI (95% CI)	p value
BMI	0.57 (0.47-0.67)	Reference		Reference	
BMI + caIMR	0.66 (0.57-0.76)	4.3 (0.1-12.3)	0.020	32.8 (0.0-55.4)	0.020

AUC, Area under curve; IDI, Integrated discrimination improvement; NRI, Net reclassification index; CI, confidence interval; BMI, body mass index; caIMR, coronary angiography−derivedindex of microcirculatory resistance.

## Discussion

The present investigation is the first to evaluate the prognostic value of the coronary microvascular function in overweight with CCS patients reflected by the less-invasive and pressure-wire-free caIMR index. The major findings of the present study were: 1) impaired coronary microvascular function was more common among overweight with CCS; 2) high caIMR was associated with worsening clinical outcomes, as well as had good diagnostic and prognostic performance in CCS patients with overweight. Our findings provide valuable insight into the impact of caIMR on overweight patients, given its less invasiveness, pressure-wire-free design, and no hyperemic agents.

Several epidemiologic studies have investigated the relationship between obesity, overweight, and adverse clinical outcomes (26, 27). Obesity has been implicated in increased systemic vascular resistance and increased risk of cardiovascular conditions (27, 28). Prior studies have shown that patients with central obesity are at an increased risk of atherosclerosis and poor clinical outcomes (29, 30). A study by Hubert et al. discovered that obesity is an independent risk factor for cardiovascular disease after 26-year of follow-up (31). High BMI was associated with a significantly increased risk of death among more than one million participants recruited in Asia (6). An elevated BMI is also strongly linked to an increased mortality rate, according to the largest cohort study with 363,2674 participants (32).

Besides microvascular angina, CMD is also involved in several cardiac and systemic diseases; emerging evidence suggests a pathogenic role of cardiovascular risk factors such as diabetes, hypertension, metabolic syndrome, and obesity in the pathogenesis of CMD (33, 34). CMD has recently been found to be more common in obese patients. An investigation using 13N-ammonia PET-MPI reported that 43.2% of non-centrally obese patients and 53.7% of centrally obese patients had CMD (12). Lee et al. reported that high IMR was associated with obesity, history of MI, right coronary artery, and female sex, suggesting that obese patients are more likely to have CMD (35). Myocardial blood flow was significantly reduced in obese and overweight individuals with normal metabolism, suggesting impaired coronary microcirculatory function (9). The interaction mechanism between obesity and CMD has been reported in previous studies indicating that adipose tissue deposition can lead to tissue hypoxia, which leads to an increase in cytokines such as leptin and interleukin-6; these adipokines and pro-inflammatory cytokines result in oxidative stress response that may lead to endothelial and microvascular dysfunction (36). In our study, we found that impaired coronary microvascular function was more common among CCS patients with overweight than normal-weight patients, which was in line with previous studies that indicate that CMD is more common among obese individuals.

As previous studies reported, CMD is closely linked with adverse cardiovascular events in multiple diseases (37, 38). The potential risk of CMD-related adverse events extends beyond obstructive coronary artery disease to risk factors such as obesity (11, 39). CMD increases in severity with increasing BMI and is a potential determinant of future cardiovascular disease risk than BMI or established risk factors (11). In a long-term study among obese patients with normal perfusion, Bajaj et al. reported that CMD was an independent predictor of adverse events, while reduced CFR was associated with a 2.5-fold increased risk of events (11). Our study extends the results of previous studies and found that the incidence of MACE in overweight patients with CMD was significantly higher than that in non-CMD patients, while there were no significant differences in normal-weight patients.

The coronary microvascular function can be assessed either invasively or using noninvasive methods, depending on the clinical situation (40). Among the invasive techniques, CFR and IMR are widely used wire-based measures of coronary microvascular function (16, 40). Invasive IMR as a quantitative assessment of the hyperemic microvascular resistance has shown better reproducibility even in patients with CCS (41). Earlier studies showed that IMR is a better indicator of impaired microvascular function than other measures among various patient populations, including patients with the acute coronary syndrome (42). Notably, in a multicenter prospective study among CAD patients, Lee et al. reported that obesity was strongly associated with high IMR, which suggests that obesity is potentially linked with CMD and cardiovascular risk (35). Although invasive IMR is a strong predictor of short and long-term cardiac events, unfortunately, due to some practical limitations, its use has been limited mainly due to IMR requiring coronary pressure-sensor guidewires and adenosine-induced hyperemia, complex operation, high cost, and long measurement time. caIMR is a new simple technique for assessing the state of the coronary microcirculation without a coronary guidewire and pharmacologic hyperemia (17). Several studies in different patient populations have reported good diagnostic and prognostic ability of caIMR. In a recent study among patients with non-obstructive coronary artery disease, caIMR significantly correlated with IMR to diagnose microcirculatory dysfunction (17). Choi et al. showed that the caIMR was significantly associated with adverse cardiac events in STEMI patients (19). In patients with CAD, Dai N et al. (21) reported that caIMR measured after PCI predicts the risk of cardiac death or readmission due to heart failure. Our recent study also found that caIMR had an excellent prognostic ability for MACE among myocardial infarction with non-obstructive coronary arteries (MINOCA) patients (20). Among acute coronary syndromes and stable CAD, Scarsini et al. demonstrated similar findings and reported that caIMR correlated well with IMR to predict microvascular obstruction (43). However, the prognostic impact of IMR among obese patients has not been investigated, regardless of invasive or non-invasive. To the best of our knowledge, the present study is the first to evaluate the prognostic significance of caIMR in overweight patients. Our study found that a high caIMR is an independent predictor for worse clinical outcomes in overweight patients but not in patients with normal weight. Importantly, patients with high caIMR had a significantly higher rate of adverse events than patients with low caIMR. Notably, there was a significant effect of the interaction term of high caIMR in predicting MACE between overweight and normal-weight groups; this result better supports our conclusion that calMR≥25 was associated with an increased risk of MACE only in overweight patients but not in normal-weight patients. Further research studies with a large sample size are needed to confirm the present study’s findings.

Taken together, the findings of the present investigation are of significant clinical interest, which highlight a population at higher risk for overt cardiac dysfunction and adverse prognosis. caIMR may provide additional information on high-risk individuals and help manage or prevent adverse outcomes. Meanwhile, caIMR could potentially reduce the use of a pressure wire, reduce technical error, and enhance the adoption of physiological assessment of coronary microvascular function.

### Limitations

Several limitations were presented in this study. First, this was a single-center study, and on account of the small sample size, the findings may not be generalizable to a broad population. Second, caIMR was measured based on the angiography-derived diastolic flow velocity in contrast-induced sub-hyperemia. Third, in this study, we employed BMI≥24.0kg/m² to identify overweight among Chinese populations; thus, the findings of our study may not apply to populations of different ethnic backgrounds with body surface area; future studies are required to evaluate the association between caIMR and outcomes in overweight patients using different definitions. Fourth, the longer follow-up and the comparison before and after regular pharmacotherapy or bariatric surgery need further examination. In addition, in our study, we did not measure more indicators that reflect the fat distribution.

## Conclusion

Impaired coronary microvascular function assessed by caIMR was common and is an independent predictor of MACE among overweight patients with CCS. The evaluation of caIMR can provide an objective risk stratification method for patients with overweight and could potentially enhance the adoption of physiological assessment of coronary microvascular function.

## Data availability statement

The raw data supporting the conclusions of this article will be made available by the authors, without undue reservation.

## Ethics statement

The studies involving human participants were reviewed and approved by Shanghai Tenth People’s Hospital, Tongji University. The patients/participants provided their written informed consent to participate in this study.

## Author contributions

CF, FA, XY and WC designed the study. WZ, LL, AQM, and GY collected the data. CF, FA, FY, AAM, RMM, XL, TS, YX and WZ were involved in data cleaning, follow-up, and verification. CF, FA, and WC drafted the manuscript and revised it critically for important intellectual content. WC and XY approved the final version of the manuscript. All authors contributed to manuscript revision, read, and approved the final version.

## Funding

This work was supported by Chinese National Natural Science Foundation (82170521), Shanghai Natural Science Foundation of China (21ZR1449500), Foundation of Shanghai Municipal Health Commission (202140263), the Fundamental Research Funds for Central Universities (NO.22120190211), Foundation of Chongming (CKY2021-21, CKY2020-29), Clinical Research Plan of Shanghai Tenth People’s Hospital (YNCR2A001), and Clinical Research Plan of SHDC (SHDC2020CR4065).

## Acknowledgments

Those who contributed to this work and met the authorship criteria are listed as authors of this manuscript.

## Conflict of interest

The authors declare that the research was conducted in the absence of any commercial or financial relationships that could be construed as a potential conflict of interest.

## Publisher’s note

All claims expressed in this article are solely those of the authors and do not necessarily represent those of their affiliated organizations, or those of the publisher, the editors and the reviewers. Any product that may be evaluated in this article, or claim that may be made by its manufacturer, is not guaranteed or endorsed by the publisher.

## Abbreviations

AUC, area under the curve; BMI, body mass index; CAD, coronary heart disease; caFFR, coronary angiography-derived fractional flow reserve; CAG, coronary angiography; caIMR, coronary angiography-derived index of microcirculatory resistance; CCS, chronic coronary syndrome; CFR, coronary flow reserve; CI, confidence interval; CMD, coronary microvascular dysfunction; HbA1c, hemoglobin A1c; HDL, high density lipoprotein; HR, hazard ratio; IMR, index of microcirculatory resistance; LAD, left anterior descending artery; LCX, left circumflex artery; LDL, low density lipoprotein; LVEF, left ventricular ejection fraction; MACE, major adverse cardiac events; MI, myocardial infarction; MINOCA, myocardial infarction with non-obstructive coronary arteries; PCI, percutaneous coronary intervention; RCA, right coronary artery; ROC, receiver operating characteristic; STEMI, ST-Segment elevation myocardial infarction; TC, total cholesterol; TG, triglycerides.
